# Effects of prebiotics on sepsis, necrotizing enterocolitis, mortality, feeding intolerance, time to full enteral feeding, length of hospital stay, and stool frequency in preterm infants: a meta-analysis

**DOI:** 10.1038/s41430-018-0377-6

**Published:** 2018-12-19

**Authors:** Cheng Chi, Nicholas Buys, Cheng Li, Jing Sun, Chenghong Yin

**Affiliations:** 1 Department of Internal Medicine, Beijing Obstetrics and Gynecology Hospital, Capital Medical University, Beijing, China; 2 Menzies Health Institute Queensland, Griffith University, Gold Coast, QLD Australia; 3 School of Medicine, Griffith University, Gold Coast, Australia

**Keywords:** Risk factors, Infectious diseases

## Abstract

**Background/Objectives:**

Prebiotics are increasingly recognized as an effective measure to promote health and prevent adverse health outcomes in preterm infants. We aimed to systematically review the randomized controlled trials (RCTs) in this area.

**Subjects/Methods:**

Relevant studies from January 2000 to June 2018 were searched and selected from PubMed, Medline, Scopus, and the Cochrane Library. RCTs were included if they involved preterm infant participants, included a prebiotic intervention group, measured incidence of sepsis, feeding intolerance, mortality, time to full enteral feeding, necrotizing enterocolitis (NEC), length of hospital stay, and stool frequency as outcomes.

**Results:**

Eighteen RCTs (*n* = 1322) were included in the final meta-analysis. Participants who took prebiotics showed significant decreases in the incidence of sepsis (with a risk ratio (RR) of 0.64, 95% CI: 0.51, 0.78), mortality (RR = 0.58. 95% CI: 0.36, 0.94), length of hospital stay (mean difference (MD): −5.18, 95% CI: −8.94, −1.11), and time to full enteral feeding (MD: −0.99, 95% CI: −1.15, 0.83). The pooled effects showed no significant differences between intervention and control groups in relation to the morbidity rate of NEC (RR = 0.79, 95% CI: 0.44, 1.44) or feeding intolerance (RR = 0.87, 95% CI: 0.52, 1.45).

**Conclusions:**

The results showed that the use of prebiotics with preterm infants is safe and can decrease the incidence of sepsis, mortality, length of hospital stay, and time to full enteral feeding but not NEC.

## Introduction

Due to advances in medical technology, outcomes for preterm infants treated in neonatal intensive care units (NICUs) have improved during the latest 20 years [1]. However, more than 40% of very preterm infants still die before discharge or suffer from one or more serious complications [2] that can lead to further poor neurodevelopmental outcomes [3]. Most preterm infant deaths, especially those born<28 weeks gestation, were caused by infections, including sepsis and necrotizing enterocolitis (NEC). These morbidities are associated with increased mortality, morbidity, and prolonged lengths of hospital stay [3, 4].

The gut microbial composition of preterm infants is quite different from that of full-term infants. Preterm infants have low bacterial diversity and a different gut microbiota, with more Proteobacteria and *Enterococcus*, which are regarded as potential pathogenic bacteria in their intestinal tract [5]. In addition, the digestive system of preterm infants is immature, with an underdeveloped intestinal mucosa barrier [6]. Pathogens and bacterial toxins can pass through their gut barrier easily and enter blood and lymph circulation, leading to life-threatening infections, including sepsis, NEC, and diarrhea. There are many factors that may lead to intestinal dysbacteriosis of preterm infants, such as lack of fresh breast milk, delayed introduction of enteral feeding, and excessive antibiotic use in the NICU [6, 7]. After birth, most preterm infants stay in the hospital to receive observation and treatment for at least 1–2 weeks, which can expose them to a higher incidence of opportunistic infections [8].

In recent years, much attention has been given to providing preterm infants with prebiotics supplements to promote growth and development and prevention of various morbidities. Prebiotics supplementation in preterm infants may facilitate the growth and proliferation of probiotic bacteria in their intestinal tract and has been found to prevent the overgrowth of pathogens and promote the maturation of the intestinal mucosa. However, due to their immature immune and digestive systems [9], prebiotic supplementation dosage levels should be considered carefully to avoid feeding intolerance.

Non-human milk oligosaccharides, which have been manufactured to function in a similar manner to oligosaccharides in breast milk [7], include neutral short-chain galacto-oligosaccharides (scGOS), long-chain fructo-oligosaccharides (lcFOS), and pectin-derived acidic oligosaccharides (pAOS). In addition to neutral oligosaccharides (scGOS/lcFOS), few clinical trials are concerned with acidic oligosaccharides (pAOS), which could directly modify the immune system of infants [7].

Many meta-analyses focused on evaluating if probiotics have shown beneficial effects in preterm infants, but few have focused on the effect of prebiotics. Furthermore, there are no meta-analyses that evaluate the effect of pAOS in relation to preterm infants. We aimed to perform a meta-analysis of studies published over the past 18 years, in which randomized control trials (RCTs) were used to evaluate whether prebiotics could confer a health benefit to preterm infants, especially in reducing the incidence of sepsis, NEC, and mortality. Furthermore, this study also considered other important outcomes in preterm infants, including prolonged length of hospital stay, feeding intolerance, and stool frequency.

## Methods

### Search strategy

The study protocol was registered in the PROSPERO database (registration ID: CRD42017068320). The PICO approach was used to assist in identifying relevant studies as follows: P (population): study population comprising preterm or low birth weight infants, and all trials involved human participants; I (intervention): use one of the following supplements as intervention: scGOS, lcFOS, pAOS, oligosaccharides, fructans, inulin, or oligofructose as intervention; C (comparison): placebo-controlled trials involved participants randomized allocation to treatment groups; O (outcome): report one or more of the following outcomes: morbidity rate of sepsis, NEC, mortality, feeding intolerance, time to achieve full enteral feeding, stool frequency, and length of hospital stay. If the authors published multiple articles from the same population, we only chose those with the largest sample sizes and the longest intervention duration.

The following items were combined and used to conduct a systematic search to identify suitable trials: prebiotics AND preterm infant or low weight birth infant AND search filters for RCTs. When relevant data was not adequately provided in articles, we contacted the authors by e-mail in an attempt to retrieve the missing information needed for the meta-analysis.

### Inclusion criteria

We used the following criteria to determine the inclusion of RCTs: (1) Published in a peer-reviewed journal in the past 18 years (January 2000 until June 2018); (2) Study design was a RCT; (3) Participants included low birth weight infants (<2500 g) or preterm infants (<36 weeks); (4) Prebiotics and placebo were supplied to different groups as an intervention; and (5) Outcome variables included incidence of sepsis, NEC, mortality, time to achieve full enteral feeding, feeding intolerance, stool frequency, and length of hospital stay.

### Data extraction and quality assessment

**Fig. 1:**
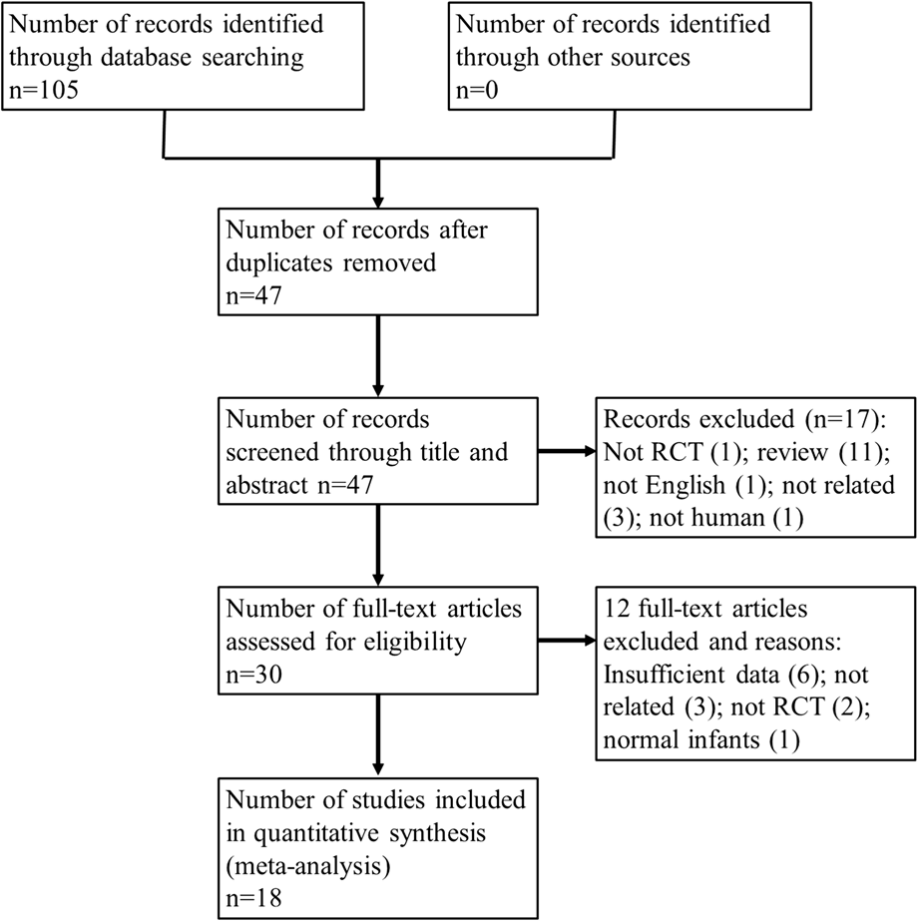
The preferred reporting items for systematic reviews and meta-analyses flow chart representing the reviewing process

Two researchers independently extracted data and conducted an assessment of trials according to the criteria above. Relevant studies from January 2000 to June 2018 were searched and selected from PubMed, Medline, Scopus, and the Cochrane Library. A summary of the review was presented using the PRISMA flow chart (Fig. 1). We extracted the following data from each of the included articles: study location, population, study design, type of prebiotics, and placebo used as the intervention, details of the main study endpoints, blinding, form and dosage of prebiotics used, duration, information to assess the risk of bias, and major outcomes reported. We used the Physiotherapy Evidence Database (PEDro) tool to evaluate the quality of the included literature. Studies were then categorized into three levels using the tool (high, 8 or more points; moderate, 4–7 points; low, 3 points or less). Studies that were categorized as moderate or high quality were included in the analysis, while poor studies were excluded.

### Statistical analysis

A pooled effect size method was used to describe the effect of prebiotics on health outcome variables. We used random effect models to assess the between-study variation. Risk ratios were calculated to examine the effect size for sepsis, NEC, mortality, and feeding intolerance. Mean difference was used to present the effect of time to achieve full enteral feeding, stool frequency as well as length of hospital stay. The pooled mean difference with a 95% Confidence Interval (CI) was figured up to assess the effects of prebiotics on the infants’ health outcomes. Several methods were used to estimate heterogeneity among the pooled studies, including *I*^2^ statistics as well as visual inspection of CI overlap. Low heterogeneity was defined as trials with *I*^2^ < 50%. We performed a subgroup analysis to identify the sources of heterogeneity associated with the effects of birth weight (<1500 g compared with 1500–2500 g), duration of intervention (<28 days compared with 28 days or more), quality of RCTs (high compared with moderate), the forms of prebiotics used (milk compared with other forms), dosage of prebiotics (<1.5 g/kg/day vs. 1.5 g/kg/day), and type of prebiotics (pAOS compared with no pAOS). The Egger test was used to assess any publication bias together with a visual inspection of funnel plots. Sensitivity analyses were performed to assess whether the efficacy of prebiotics was derived from one particular trial or multiple trials.

## Results

In the initial systematic search, 105 articles were identified from the databases listed above (Fig. 1). After duplications were removed, 47 articles were listed. After reading titles and abstracts, 30 potentially relevant articles were identified. Full articles were retrieved and reviewed, then another 12 studies were excluded (six due to insufficient data, four due to not focusing on preterm infants, two due to being non-human population RCT’s). The final statistical analysis was conducted with the remaining 18 articles, which consisted of 12 high quality (8 points or more) and six moderate quality (4–7 points) articles.

The 18 RCTs reported health outcomes on 647 infants given prebiotics and 675 infants as control. Samples were drawn from ten different locations, including Iran [10–12], Netherlands [13–18], Turkey [19], Greece [20, 21], Finland [22], France [23], Israel [24], England [25], Germany [26], and Italy [27]. Among the 18 trials, 11 were double-blinded [11, 12, 14, 15, 19, 21–26], and the rest seven were single blinded [10, 16–18, 20, 27]. All studies included one or more of the following outcomes: incidence of sepsis, NEC, death, time to full achieve enteral feeding, feeding intolerance, stool frequency, and length of time in hospital. The characteristics of included trials and quality assessment results are presented in Table 1.

**Table 1:** Characteristics of 18 included randomized controlled trials

Studies	Participants at beginning, *n* (P/C)	Design, location	Gestational age, P/C, week	Birth weight, P/C, g	Sex, *n* (P/C, M/F)	Treatment duration, days	Prebiotics used	Dose	Placebo	Measured outcomes	Key outcomes	Quality of studies assessed by PEDro tool	Form
Armanian et al. [11]	50 (25/25)	DB, Iran	30.48 (2.31)/29.80 (2.16)	1.262 (0.213)/1.188 (0.194)	Not mentioned	21	scGOS/lcFOS	1.5 g/kg/day	Distilled water	Fecal microbiota pattern, duration of dependency to oxygen, hospitalization, and death	Led to the rapid growth of beneficial Lactobacillus colonies	11	Mixture solution
Van den Berg et al. [18]	77 (38/39)	SB, Netherlands	29.9 (1.7)/29.6 (2.1)	1.32 (0.38)/1.28 (0.28)	21/17, 24/15	28	scGOS/lcFOS/pAOS	1.5 g/kg/day	Breast milk or formula	Neurodevelopment, cytokines, and infections	No significant improvement of neurodevelopmental outcomes	8	With breast milk or formula
Armanian et al. [12]	50 (25/25)	DB, Iran	30.4 (2.3)/29.8 (2.1)	1.262 (0.213)/1.188 (0.194)	Not mentioned	7	scGOS/lcFOS	1.5 g/kg/day	Distilled water	Bilirubin level and stool frequency	Increase stool frequency, improve feeding tolerance, and reduce bilirubin level	11	Mixture solution
Dilli et al. [19]	200 (50/50)	DB, Turkey	29.0 (1.7)/28.2 (2.2)	1.229 (0.246)/1.147 (0.271)	52/48, 58/42	56	Inulin	1.35 g/kg/day	Maltodextrin	NEC, sepsis, mortality, duration of hospital	Inulin could not decrease NEC	10	With breast milk or formula
Armanian et al. [10]	75 (25/50)	SB, Iran	30.48 (2.31)/30.38 (2.53)	1.263 (0.213)/1.206 (0.177)	Not mentioned	14	scGOS/lcFOS	1.5 g/kg/day	Distilled water	NEC, mortality, sepsis, feeding intolerance, and days to reach full enteral feeding	Lower NEC, mortality, sepsis rates, and shorter days to reach full enteral feeding	8	With breast milk or formula
Dasopoulou et al. [21]	167 (85/82)	DB, Greece	34.0 (0.33)/34.0 (0.33)	2.019 (0.30)/1.987 (0.38)	Not mentioned	16	scGOS/lcFOS	1.2 g/kg/day	Formula	Motilin, NEC, mortality, sepsis, and feeding intolerance	Increase motilin, reduce gastric residue	11	With formula
Luoto et al. [22]	47 (23/24)	DB, Finland	32–35	2.123 (0.39)/2.412 (0.84)	11/12, 19/5	57	scGOS/polydextrose	1.2 g/kg/day	Microcrystalline cellulose and dextrose anhydrate	Respiratory tract infections and its duration	Reduce the risk of rhinovirus infections	11	With breast milk or formula
Van den Berg et al. [16]	103 (55/58)	SB, Netherlands	29.9 (1.9)/29.3 (2.1)	1.3 (0.4)/1.2 (0.3)	31/24, 36/22	28	scGOS/lcFOS/pAOS	1.5 g/kg/day	Maltodextrin	Serious infectious morbidity	Does not improve the immunization response	10	With breast milk or formula
LeCouffe et al. [17]	113 (48/45)	SB, Netherlands	30.2 (1.6)/29.5 (2.0)	1.37 (0.4)/1.26 (0.3)	26/48, 26/45	28	scGOS/lcFOS/pAOS	1.5 g/kg/day	Maltodextrin	Neurodevelopmental outcome	No effect on neurodevelopmental	8	With breast milk or formula
Niele et al. [15]	114 (48/46)	DB, Netherlands	30.1 (1.6)/29.5 (2.0)	1.40 (0.4)/1.30 (0.3)	Not mentioned	28	scGOS/lcFOS/pAOS	1.5 g/kg/day	Maltodextrin	Allergic and infectious diseases	Does not decrease the incidence of allergic and infectious diseases	10	With breast milk or formula
Westerbeek et al. [14]	103 (55/58)	DB, Netherlands	29.9 (1.9)/29.3 (2.1)	1.3 (0.4)/1.2 (0.3)	31/24, 36/22	28	scGOS/lcFOS/pAOS	1.5 g/kg/day	Maltodextrin	Stool viscosity, stool frequency, and stool pH	Decreases stool viscosity and stool pH, increased stool frequency	11	With breast milk or formula
Campeotto et al. [23]	58 (24/34)	DB, France	33.5 (1.3)/33.4 (1.1)	1.91 (0.35)/1.93 (0.39)	15/9, 16/18	30	Fermente induced non-digestible oligosaccharides	Not mentioned	Formula	Inflammatory and immune markers	Benefits on inflammatory and immune markers	10	Formula
Riskin et al. [24]	28 (15/13)	DB, Israel	30.3 (2.8)/28.7 (2.9)	1.52 (0.55)/1.21 (0.45)	10/5, 5/8	35	Lactulose	1.5 g/kg/day	Dextrose	NEC, mortality, sepsis, feeding intolerance, and days to reach full enteral feeding	Suggest positive prebiotic effects	10	Mixture solution
Modi et al. [25]	154 (73/81)	DB, UK	30.0 (0.5)/31.0 (0.5)	1.57 (0.88)/1.52 (0.91)	48/25, 50/31	28	scGOS/lcFOS	1.2 g/kg/day	Formula	NEC, mortality, sepsis, feeding intolerance	Safe and may benefit enteral tolerance	11	Formula
Kapiki et al. [20]	56 (36/20)	SB, Greece	33.4 (1.8)/33.9 (1.3)	1.59 (0.33)/1.64 (0.17)	16/17, 8/12	7	scGOS	0.6 g/kg/day	Maltodextrin	Bifidogenic effect, stool characteristics	Small quantity of prebiotic is well accepted	7	Formula
Mihatsch et al. [26]	20 (10/10)	DB, Germany	27.1 (2.2)/27.6 (3.3)	0.89 (0.64)/0.90 (0.69)	Not mentioned	14	scGOS/lcFOS	1.5 g/kg/day	Maltodextrin	Stool characteristics	Reduce stool viscosity	11	Formula
Boehm et al. [27]	27 (12/15)	SB, Italy	31.2 (0.6)/31.4 (0.9)	1.59 (0.24)/1.60 (0.32)	8/7, 7/5	28	scGOS/lcFOS	1.5 g/kg/day	Maltodextrin	Stool characteristics	More frequent and softer stools	8	Formula

Values are means (SDs) unless otherwise indicated

*scGOS/lcFOS* short chain galacto-oligosaccharides/long chain fructo-oligosaccharides, *pAOS* pectin-derived acidic oligosaccharides, *DB* double blind, *SB* single blind, *P/C* prebiotic group/control group

### Effects on sepsis

**Fig. 2:**
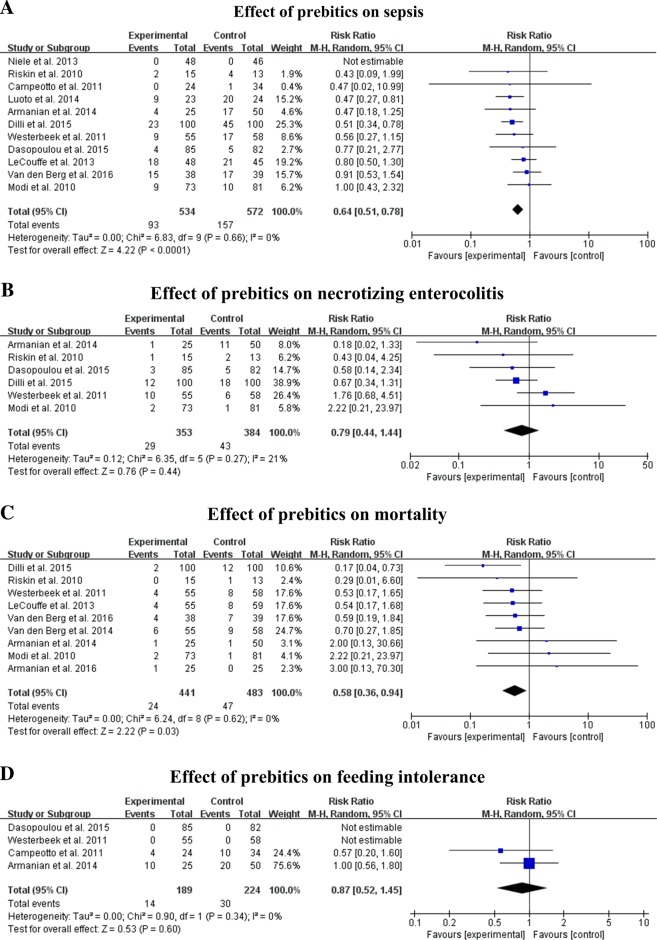
Forest plots of the effects of prebiotics on sepsis (**a**), necrotizing enterocolitis (**b**), mortality (**c**), and feeding intolerance (**d**)

Eleven trials, with 1106 infants, reported sepsis rates. The mean morbidity rate for the infants receiving prebiotics was 17.4% compared with 27.4% in the controls. The results of meta-analysis showed a significant decrease in risk ratio of 0.64 (95% CI: 0.51, 0.78; *P* < 0.001) in the group of preterm infants receiving prebiotic treatment compared with that of the control group, with no significant heterogeneity among studies (*I*^2^ = 0%, *P* = 0.66) (Fig. 2a).

Subgroup analysis (Table 2) showed that prebiotics supplementation could significantly reduce the incidence of sepsis when treatment duration was ≥28 days (*P* < 0.01) compared with <28 days (*P* = 0.15). It is notable that trials that supplied prebiotics using breast milk plus preterm formula milk (*P* < 0.001) as the medium achieved better results in lowering sepsis risk compared with trials using distilled water (*P* = 0.28). It was also found that the effect of prebiotics on reducing sepsis was statistically significant when the quality assessed to be high (*P* < 0.001) compared with moderate quality ones (*P* = 0.17). A significant beneficial effect was also demonstrated in studies using prebiotics with pAOS (*P* < 0.001) compared with prebiotics without pAOS (*P* = 0.13).

**Table 2:** Subgroup analysis on the effects of probiotics on sepsis, necrotizing enterocolitis, and mortality

Subgroups	Sepsis						Necrotizing enterocolitis						Mortality					
	Studies, *n*	Participants, *n*	*I*^2^	Risk ratio	95% CI	*P*	Studies, *n*	Participants, *n*	*I*^2^	Risk ratio	95% CI	*P*	Studies, *n*	Participants, *n*	*I*^2^	Risk ratio	95% CI	*P*
Birth weight						0.66						0.92						0.54
<1500 g	6	652	4%	0.65	0.51, 0.84***		3	388	61%	0.77	0.29, 2.06		3	742	0%	0.56	0.34, 0.91*	
1500- 2500 g	5	454	0%	0.59	0.39, 0.88**		3	349	0%	0.71	0.25, 2.07		3	182	3%	1.04	0.15, 7.15	
Duration of intervention						0.76						0.18						0.17
<28 days	2	242	0%	0.64	0.51, 0.80***		2	242	0%	0.39	0.13, 1.24		2	125	0%	2.38	0.30, 18.75	
≥28 days	9	864	0%	0.63	0.51, 0.78***		4	495	17%	0.97	0.51, 1.83		4	771	0%	0.54	0.33, 0.90*	
Form of prebiotics						0.62						0.6						0.68
Milk	10	1078	0%	0.64	0.52, 0.79***		5	709	34%	0.82	0.42, 1.62		5	846	0%	0.57	0.35, 0.93*	
Other	1	28	Not applicable	0.43	0.09, 1.99		1	28	Not applicable	0.43	0.04, 4.25		1	78	6%	0.92	0.09 9.07	
Quality of study						0.1						0.13						0.83
High	8	861	0%	0.55	0.42, 0.72***		5	662	0%	0.88	0.54, 1.43		5	658	8%	0.56	0.29, 1.07	
Moderate	3	245	0%	0.79	0.57, 1.11		1	75	Not applicable	0.18	0.02, 1.33		1	266	0%	0.62	0.29, 1.35	
Dosage						0.2						0.92						0.87
<1.5 g/kg/day	4	568	0%	0.55	0.41, 0.75***		3	521	0%	0.7	0.39, 1.26		3	354	70%	0.51	0.04, 6.34	
1.5 g/kg/day	6	480	0%	0.73	0.54, 0.98*		3	216	60%	0.64	0.14, 3.02		3	570	0%	0.64	0.38, 1.07	
Type of prebiotics						0.09						0.06						0.83
Without pAOS	4	377	0%	0.78	0.57, 1.07		5	624	0%	0.67	0.36, 1.06		5	507	34%	0.7	0.18, 2.65	
With pAOS	7	729	0%	0.54	0.41, 0.72***		1	113	Not applicable	1.76	0.68, 4.51		1	417	0%	0.59	0.34, 1.02	

*P < 0.05, **P < 0.01, ***P < 0.001

### Effects on NEC

Six trials, with 737 infant included, presented the morbidity rate of NEC. The mean NEC rate in the prebiotics intervention and control group was 8.2% and 11.2%, respectively. The meta-analysis showed a non-significant risk ratio of 0.79 (95% CI: 0.44, 1.44; *P* = 0.44) between the two groups administered prebiotics and placebo. The heterogeneity of trials was not significant (*I*^2^ = 21%, *P* = 0.27) (Fig. 2b). No significant difference was found between the two groups in morbidity rate of NEC, considering birth weight, treatment duration, form of prebiotics, quality of studies, dosage, and the administration of pAOS (Table 2).

### Effects on mortality

Nine trials, including 924 participants, reported the mortality rates of preterm infants. The mean death rate in the infants receiving prebiotics was 5.4% vs. 9.7% in the controls. The meta-analysis showed a significantly lower risk ratio of 0.58 (95% CI: 0.36, 0.94; *P* < 0.001) in the prebiotics intervention infants compared with the infants receiving the placebo. The heterogeneity of trials was not significant (*I*^2^ = 0%, *P* = 0.62) (Fig. 2c).

Subgroup analysis (Table 2) showed that the prebiotics supplementation could significantly reduce the morbidity rate of sepsis when treatment duration was 28 days or more (*P* < 0.05) compared with infants receiving prebiotics less than 28 days (*P* = 0.41). The trials administering prebiotics, along with a combination of breast milk and preterm formula (*P* < 0.05), had a greater effect on mortality compared with trials that used distilled water (*P* = 0.95). The results also showed that prebiotics treatment had a greater effect on reducing the death rate in trials with high quality (*P* < 0.001) compared with that of moderate quality (*P* = 0.17). In addition, prebiotics treatment had a significant effect on preterm infants with a birth weight less than 1500 g (*P* < 0.05) but not with preterm infants with a birth weight ≥1500 g (*P* = 0.97).

### Effects on feeding intolerance

Four trials, involving 413 infants, reported the incidence of infant feeding intolerance. The mean feeding intolerance rate in infants administered prebiotics was 7.4% compared with 13.4% in controls. There was a non-significant risk ratio of 0.87 (95% CI: 0.52, 1.45; *P* = 0.60) between the infants receiving the prebiotics intervention compared to the placebo. The heterogeneity of trials was not significant (*I*^2^ = 0%, *P* = 0.34) (Fig. 2d). No significant difference in reducing the incidence of feeding intolerance was found by subgroup analysis, considering birth weight, treatment duration, form of prebiotics, quality of studies, dosage, and the administration of pAOS.

### Effects on time to achieve full enteral feeding

**Fig. 3:**
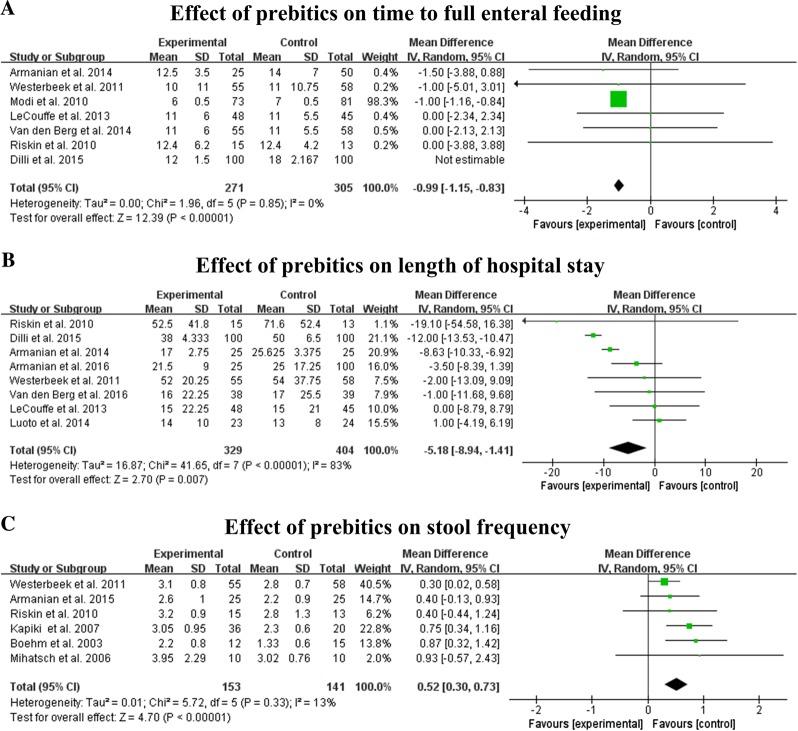
Forest plots of the effects of prebiotics on time to full enteral feeding (**a**), length of hospital stay (**b**), and stool frequency (**c**)

Six studies, including 576 infant participants, measured time to achieve full enteral feeding. The results of meta-analysis showed a significantly shorter time to achieve full enteral feeding in the prebiotic group when compared with the control group (MD −0.99, 95% CI: −1.15, 0.83, *P* < 0.001). The heterogeneity of included trials was not significant (*I*^2^ = 0%, *P* = 0.85) (Fig. 3a). Subgroup analysis (Table 3) indicated that trials that administered prebiotics along with a combination of human breast milk and preterm formula (*P* < 0.05) tended to be more effective compared with the trial using distilled water (*P* = 1). The results also showed that the effect of prebiotics on reducing time to full enteral feeding was statistically significant in trials of high quality (*P* < 0.001) compared to trials of moderate quality (*P* = 0.39). One study [10] with a 14-day intervention showed no significant difference between groups using prebiotics or not (*P* = 0.22). Five studies [14, 16, 17, 24, 25] showed that the infant receiving prebiotics spent less time in achieving full enteral feeding compared to the controls (*P* < 0.01).

**Table 3:** Subgroup analysis on the effects of probiotics on time to full enteral feeding, length of hospital stay, and stool frequency

Subgroups	Time to full enteral feeding						Length of hospital stay						Stool frequency					
	Studies, *n*	Participants, *n*	*I*^2^	Mean difference	95% CI	*P*	Studies, *n*	Participants, *n*	*I*^2^	Mean difference	95% CI	*P*	Studies, *n*	Participants, *n*	*I*^2^	Mean difference	95% CI	*P*
Birth weight						0.64						0.39						0.04
<1500 g	5	594	94%	−1.8	−5.21, 1.61		6	658	80%	−6.6	−10.03, −3.08***		3	183	0%	0.34	0.10, 0.58***	
1500–2500 g	2	182	0%	−1	−1.16, −0.84		2	75	17%	−1.07	−13.05, 10.91		3	111	0%	0.74	0.44, 1.05***	
Duration of intervention						0.99						0.55						0.55
<28 days	1	75	Not applicable	−1.5	−3.88, 0.88		2	175	73%	−6.95	−11.51, −1.68***		3	126	0%	0.63	0.32, 0.95***	
≥28 days	6	701	98%	−1.47	−4.29, 1.36		6	558	85%	−3.87	−11.38, 3.64		3	168	40%	0.48	0.11, 0.86	
Form of prebiotics					0.49							0.12						0.48
Milk	6	748	98%	−1.67	−4.43, 1.08		6	655	88%	−3.4	−9.71, 2.91		4	216	46%	0.6	0.27, 0.93***	
Other	1	28	Not applicable	0	−3.88, 3.88		2	78	0%	−8.65	−10.35, −6.94***		2	78	0%	0.4	−0.05, 0.85***	
Quality of study						0.58						0.13						0.03
High	5	608	99%	−1.76	−4.92, 1.4		5	438	86%	−6.98	−11.31, −2.65***		4	211	0%	0.34	0.11, 0.58***	
Moderate	2	168	0%	−0.74	−2.4, 0.93		3	295	0%	−2.44	−6.41, 1.52		2	83	0%	0.79	0.47, 1.12***	
Dosage						0.24						0.89						0.16
<1.5 g/kg/day	2	354	100%	−3.49	−8.39, 1.41		2	247	95%	−5.75	−18.48, 6.98		1	56	Not applicable	0.75	0.34, 1.16***	
1.5 g/kg/day	5	422	0%	−0.46	−1.65, 0.73		6	486	49%	−4.8	−8.78, −0.82*		5	238	0%	0.42	0.21, 0.64***	
Type of prebiotics						0.27						0.03						0.06
Without pAOS	4	457	99%	−2.29	−5.8, 1.23		5	389	92%	−10.81	−19.62, −2.01*		5	181	0%	0.66	0.41, 0.92***	
With pAOS	3	319	0%	−0.13	−1.6, 1.33		3	283	0%	−7.16	−6.63, 4.95		1	113	Not applicable	0.3	0.02, 0.58*	

*P < 0.05, **P < 0.01, ***P < 0.001

### Effects on length of hospital stay

Eight trials, comprising 733 infant participants, reported the length of hospital stay. Data from six studies [10, 12, 14, 18, 19, 24] showed that the infants received prebiotic supplement had a shorter length of hospital stay, while two studies [17, 22] did not observe any significant difference on this variable. The meta-analysis showed a significant reduction in length of hospital stay of 0.58 (MD −5.18, 95% CI: −8.94, −1.11, *P* = 0.007) (Fig. 3b) in the prebiotics intervention infants compared with the infants receiving the placebo. The heterogeneity of trials was significant (*I*^2^ = 83%, *P* < 0.001), and subgroup analysis (Table 3) did not improve the heterogeneity. We also performed a sensitivity analysis to address the heterogeneity and confirm the stability of our results. The cumulative sensitivity test also demonstrated the total effect was not due to any single study.

### Effects on stool frequency

Six studies with 294 infant participants measured stool frequency. The stool frequency in the prebiotic group was significantly higher when compared to the control group (MD 0.52, 95% CI: 0.3, 0.73, *P* < 0.001). The heterogeneity of trials was not significant (*I*^2^ = 13%, *P* = 0.33) (Fig. 3c). Studies with prebiotics administered via a combination of breast and formula milk (*P* < 0.001) showed a significantly higher stool frequency compared with studies that used other mediums (*P* = 0.08). In addition, subgroup analysis showed no significant difference in stool frequency, considering birth weight, treatment duration, quality of studies, and dosage (Table 3).

### Publication bias

The visual inspection of each funnel plots showed no obvious publication bias associated with the efficacy of prebiotics on infection, feeding intolerance, time to full enteral feeding, mortality, NEC, stool frequency, and length of stay in hospital. This conclusion was supported by results from the Egger’s test (sepsis: *P* = 0.80, NEC: *P* = 0.73, mortality: *P* = 0.73, time to full enteral feeding: *P* = 0.63, length of hospital stay: *P* = 0.08, stool frequency: *P* = 0.32) (Table 4), which showed no statistically significant evidence of publication bias.

**Table 4:** Publication bias illustrated by Egger test

Variable name	*t* (95% CI)	*P*
Sepsis	0.26 (−2.42, 1.93)	0.8
NEC	0.36 (−3.90, 3.99)	0.73
Mortality	0.36 (−3.90, 3.99)	0.73
Time to full enteral feeding	0.52 (−11.27, 7.49)	0.63
Length of hospital stay	2.11 (−0.33, 4.51)	0.08
Stool frequency	1.13 (−2.18, 5.16)	0.32

## Discussion

Overall, the results, based on our meta-analysis, showed that prebiotics can improve the health of preterm infants, including decreasing the incidence of sepsis and death, reducing time to achieve full enteral feeding and hospital stay, increasing the stool frequency. Besides, the effects of prebiotics on the risk of feeding intolerance and NEC were not statistically significant. Despite improvements in NICU healthcare provided by experienced neonatologists, the morbidity rate of sepsis and NEC remain high in preterm infants [28]. This is concerning because a reduction in the morbidity rate of sepsis and NEC can significantly reduce the complications and death rate associated with premature delivery.

Our results showed prebiotics supplementation could reduce the risk of NEC, which is consistent with previous published studies [29, 30]. However, unlike our study, previous reviews included less trials and had smaller sample sizes which reduced the power to assess for clinically important main outcomes. Our study included more RCT trials focused on medical complications and key variables. We also conducted subgroup analyses to reveal the effect of prebiotics with or without pAOS, and therefore, the effect of pAOS on reducing sepsis could be indicated.

The mechanism of prebiotics in decreasing the risk of sepsis and mortality might be related to preventing colonization of pathogenic bacteria and the overgrowth of opportunistic pathogens [31]. In addition, prebiotics can improve intestinal motility and intestinal permeability of preterm infants, leading to a better intestinal integrity of the epithelial surface. The combine action between the restrain of pathogens and inhibition of pathogens adhere to the epithelial surface may also be involved in promoting the resistance of preterm infants to endogenous infections [32, 33].

Subgroup analyses showed the beneficial effects of prebiotics on lowering the morbidity rate of sepsis and death might be due to the effects of preterm infants’ gestational age, prebiotic forms, and prebiotic types (added pAOS). Potential mechanisms by which prebiotics protect preterm infants from high risk of sepsis may relate to increasing the barrier to prevent the pathogenic bacteria and toxins from migrating across the intestinal mucosa, promoting a competitive exclusion of potential pathogens. Furthermore, prebiotics could modify hosts’ response to bacterial toxins and enhance their immune responses.

In our study, we found newborns with a gestational age <28 weeks would benefit less from prebiotic supplementation. The immune system, intestinal mucosa barrier as well as a distinct gut microbiota of these very low birth weight infants are less developed [5]. Due to excessive antibiotic use in these high-risk infants, antibiotic treatment before and after birth may have dramatically affected the composition of their gut microbiota, and caused intestinal dysbacteriosis [34–36]. The imbalance needs to be addressed through the use of probiotics [37] or synbiotics [38–40]. Using prebiotics alone may do little to re-establish micro-ecological balance after excessive antibiotic exposure [41–43]. Most of the trials included in our study supplemented prebiotics by adding oligosaccharide into breast milk or formula [10, 15, 17–19, 21–23], while other trials used distilled water as a medium [11, 12, 24]. In our study, trials using breast milk or formula as a medium to supply prebiotics had significant pooled effects on reducing sepsis. This effect is in line with the ability of pAOS, which are designed to act as receptors-analogs, preventing pathogens from adhering to the epithelial surface of the digestive tract [44]. The heterogeneity among the trials included was not significant, indicating that the effect of breast milk intake on lowering the morbidity rate of sepsis was consistent. Beyond nutritional components, breast milk contains some important bioactive substances such as microbes, oligosaccharides, cytokines, immunoglobulins, and proteins, which directly influence the development of infants and shape their intestinal microbiota colonization [45]. These bioactive substances are considered not only protective but also stimulate the development and maturation of the immature immune system [46]. This could explain why we observed better results in trials using a combination of breast milk or formula as a medium to supply prebiotics rather than trials that used distilled water.

Healthcare for preterm infants face many problems in feeding, because of the increased morbidity rate of NEC and feeding intolerance, full enteral feeding is often difficult. The pooled effects based on our meta-analysis showed a notable reduced time to enteral feeding with the administration of prebiotics to preterm infants. Prebiotic supplement increases the abundances of prebiotics, such as *bifidobacteria* and *lactobacillus*, and reduce the abundance of potential pathogenic bacteria in gut microbiota of preterm infants [47, 48]. The colonization of beneficial bacteria can improve stool frequency and consistency, which in turn promote the enteral feeding tolerance and shorten the time to achieve full enteral feeding in preterm infants [26].

There were no significant differences on two items, including the morbidity rate of NEC and feeding intolerance. The morbidity of NEC could be explained by active medical intervention in the NICUs addressed infection risks such as proper fasting and parenteral nutrition [3]. The effectiveness of early detection and prevention of NEC varied from rural hospitals with relative poor medical technology to teaching hospitals in cities [2, 9]. Most of the 18 trials were conducted in teaching hospitals, in which early diagnosis and prophylactic treatment can be made by experienced clinicians. The lack of effect of prebiotics on reducing the development of NEC could also be partially due to the fact that oligosaccharides employed in the trials (scGOS, lcFOS, and pAOS) have completely different structures compared to the oligosaccharides of human milk [46], especially when they are applied to preterm infants who have a different microbiota system than full-term infants [49]. In consideration of the immature gastrointestinal tract and immune system of preterm infants, prebiotic supplementation should be administered with caution [9]. With regards to feeding intolerance, subgroup analysis suggests that prebiotics used in these trials were well tolerated by preterm infants. According to two studies [14, 16], the maximum supplementation dose of 1.5 g/kg/day appears to be effective and safe.

### Limitation

The limitation of our study is that all the variables reflect the micro-ecological balance in intestinal flora indirectly. Some early trials measured the number of bacterial colonies by cultivation which is subject to error and is potentially heterogeneous. With the development of second-generation sequencing, further RCTs are required to clarify the stains and their proportion in stool using 16S RNA sequencing or metagenomic sequencing. Only in this way can we reveal the mechanism of prebiotics on intestinal flora and preterm infant’s health directly.

### Clinical implications

The results of our study showed that prebiotics could provide significant benefits for preterm infant, including reductions of the incidence of sepsis, mortality, and time to achieve full enteral feeding. Considering these outcomes, the prebiotics supplement in NICUs would be highly beneficial for preterm infants. Prebiotics administration did little in reducing NEC and sepsis in very preterm infants. A new approach in gut microbiota management of infants is the administration of synbiotics, by combining the effect of probiotics and prebiotics. The growth of added live beneficial bacteria (probiotics) may be stimulated by specific substrates (prebiotics), which could improve the survival rate of the probiotics and provide readily substrates for fermentation. As one of the non-human milk oligosaccharides, pAOS have been produced to mimic human milk oligosaccharides. The Th2-type immune response could be attenuated better by pAOS than neutral oligosaccharides (scGOS and lcFOS) alone [50].

Overall, the results of our study showed that prebiotics administration is effective in reducing the prevalence rates of sepsis and death, shortens the time to achieve full enteral feeding and hospital stay, and increases the stool frequency. Prebiotics supplement is more effective on infants ≥28 weeks, especially when administered in accompany with breast milk plus formula, added pAOS. Prebiotics may provide a novel approach to reduce the high incidence of complications caused by sepsis and improve the health of preterm infants.

## Supplementary information

Checklist

## References

[1] Stoll BJ, Hansen NI, Bell EF, Walsh MC, Carlo WA, Shankaran S (2015). Trends in care practices, morbidity, and mortality of extremely preterm neonates, 1993–2012. JAMA.

[2] Horbar JD, Edwards EM, Greenberg LT, Morrow KA, Soll RF, Buus-Frank ME (2017). Variation in performance of neonatal intensive care units in the United States. JAMA Pediatr.

[3] Lin HC, Wu SF, Underwood M (2011). Necrotizing enterocolitis. N Engl J Med.

[4] Stoll BJ, Hansen N, Fanaroff AA, Wright LL, Carlo WA, Ehrenkranz RA (2002). Late-onset sepsis in very low birth weight neonates: the experience of the NICHD Neonatal Research Network. Pediatrics.

[5] Dahl C, Stigum H, Valeur J, Iszatt N, Lenters V, Peddada S (2018). Preterm infants have distinct microbiomes not explained by mode of delivery, breastfeeding duration or antibiotic exposure. Int J Epidemiol.

[6] Saleem B, Okogbule-Wonodi AC, Fasano A, Magder LS, Ravel J, Kapoor S (2017). Intestinal barrier maturation in very low birthweight infants: relationship to feeding and antibiotic exposure. J Pediatr.

[7] Eiwegger T, Stahl B, Haidl P, Schmitt J, Boehm G, Dehlink E (2010). Prebiotic oligosaccharides: in vitro evidence for gastrointestinal epithelial transfer and immunomodulatory properties. Pediatr Allergy Immunol.

[8] Jacquot A, Neveu D, Aujoulat F, Mercier G, Marchandin H, Jumas-Bilak E (2011). Dynamics and clinical evolution of bacterial gut microflora in extremely premature patients. J Pediatr.

[9] Frost BL, Modi BP, Jaksic T, Caplan MS (2017). New medical and surgical insights into neonatal necrotizing enterocolitis: a review. JAMA Pediatr.

[10] Armanian AM, Sadeghnia A, Hoseinzadeh M, Mirlohi M, Feizi A, Salehimehr N (2014). The effect of neutral oligosaccharides on reducing the incidence of necrotizing enterocolitis in preterm infants: a randomized clinical trial. Int J Prev Med.

[11] Armanian AM, Barekatain B, Hoseinzadeh M, Salehimehr N (2016). Prebiotics for the management of hyperbilirubinemia in preterm neonates. J Matern Fetal Neonatal Med.

[12] Armanian AM, Sadeghnia A, Hoseinzadeh M, Mirlohi M, Feizi A, Salehimehr N (2016). The effect of neutral oligosaccharides on fecal microbiota in premature infants fed exclusively with breast milk: a randomized clinical trial. J Res Pharm Pract.

[13] Westerbeek EA, van den Berg JP, Lafeber HN, Fetter WP, Boehm G, Twisk JW (2010). Neutral and acidic oligosaccharides in preterm infants: a randomized, double-blind, placebo-controlled trial. Am J Clin Nutr.

[14] Westerbeek EA, Hensgens RL, Mihatsch WA, Boehm G, Lafeber HN, van Elburg RM (2011). The effect of neutral and acidic oligosaccharides on stool viscosity, stool frequency and stool pH in preterm infants. Acta Paediatr.

[15] Niele N, van Zwol A, Westerbeek EA, Lafeber HN, van Elburg RM (2013). Effect of non-human neutral and acidic oligosaccharides on allergic and infectious diseases in preterm infants. Eur J Pediatr.

[16] van den Berg JP, Westerbeek EA, van der Klis FR, Berbers GA, Lafeber HN, van Elburg RM (2013). Neutral and acidic oligosaccharides supplementation does not increase the vaccine antibody response in preterm infants in a randomized clinical trial. PLoS ONE.

[17] LeCouffe NE, Westerbeek EA, van Schie PE, Schaaf VA, Lafeber HN, van Elburg RM (2014). Neurodevelopmental outcome during the first year of life in preterm infants after supplementation of a prebiotic mixture in the neonatal period: a follow-up study. Neuropediatrics.

[18] van den Berg JP, Westerbeek EA, Broring-Starre T, Garssen J, van Elburg RM (2016). Neurodevelopment of preterm infants at 24 months after neonatal supplementation of a prebiotic mix: a randomized trial. J Pediatr Gastroenterol Nutr.

[19] Dilli D, Aydin B, Fettah ND, Ozyazici E, Beken S, Zenciroglu A (2015). The propre-save study: effects of probiotics and prebiotics alone or combined on necrotizing enterocolitis in very low birth weight infants. J Pediatr.

[20] Kapiki A, Costalos C, Oikonomidou C, Triantafyllidou A, Loukatou E, Pertrohilou V (2007). The effect of a fructo-oligosaccharide supplemented formula on gut flora of preterm infants. Early Hum Dev.

[21] Dasopoulou M, Briana DD, Boutsikou T, Karakasidou E, Roma E, Costalos C (2015). Motilin and gastrin secretion and lipid profile in preterm neonates following prebiotics supplementation: a double-blind randomized controlled study. JPEN J Parenter Enter Nutr.

[22] Luoto R, Ruuskanen O, Waris M, Kalliomaki M, Salminen S, Isolauri E (2014). Prebiotic and probiotic supplementation prevents rhinovirus infections in preterm infants: a randomized, placebo-controlled trial. J Allergy Clin Immunol.

[23] Campeotto F, Suau A, Kapel N, Magne F, Viallon V, Ferraris L (2011). A fermented formula in pre-term infants: clinical tolerance, gut microbiota, down-regulation of faecal calprotectin and up-regulation of faecal secretory IgA. Br J Nutr.

[24] Riskin A, Hochwald O, Bader D, Srugo I, Naftali G, Kugelman A (2010). The effects of lactulose supplementation to enteral feedings in premature infants: a pilot study. J Pediatr.

[25] Modi N, Uthaya S, Fell J, Kulinskaya E (2010). A randomized, double-blind, controlled trial of the effect of prebiotic oligosaccharides on enteral tolerance in preterm infants (ISRCTN77444690). Pediatr Res.

[26] Mihatsch WA, Hoegel J, Pohlandt F (2006). Prebiotic oligosaccharides reduce stool viscosity and accelerate gastrointestinal transport in preterm infants. Acta Paediatr.

[27] Boehm G, Fanaro S, Jelinek J, Stahl B, Marini A (2003). Prebiotic concept for infant nutrition. Acta Paediatr Suppl.

[28] Henry MC, Moss RL (2008). Neonatal necrotizing enterocolitis. Semin Pediatr Surg.

[29] Srinivasjois R, Rao S, Patole S (2013). Prebiotic supplementation in preterm neonates: updated systematic review and meta-analysis of randomised controlled trials. Clin Nutr.

[30] Lohner S, Kullenberg D, Antes G, Decsi T, Meerpohl JJ (2014). Prebiotics in healthy infants and children for prevention of acute infectious diseases: a systematic review and meta-analysis. Nutr Rev.

[31] Tulumoglu S, Erdem B, Simsek O (2018). The effects of inulin and fructo-oligosaccharide on the probiotic properties of Lactobacillus spp. isolated from human milk. Z Naturforsch C.

[32] Vieira ADS, Bedani R, Albuquerque MAC, Biscola V, Saad SMI (2017). The impact of fruit and soybean by-products and amaranth on the growth of probiotic and starter microorganisms. Food Res Int.

[33] Di R, Vakkalanka MS, Onumpai C, Chau HK, White A, Rastall RA (2017). Pectic oligosaccharide structure–function relationships: prebiotics, inhibitors of *Escherichia coli* O157:H7 adhesion and reduction of Shiga toxin cytotoxicity in HT29 cells. Food Chem.

[34] Underwood MA, Sohn K (2017). The microbiota of the extremely preterm infant. Clin Perinatol.

[35] Zou ZH, Liu D, Li HD, Zhu DP, He Y, Hou T (2018). Prenatal and postnatal antibiotic exposure influences the gut microbiota of preterm infants in neonatal intensive care units. Ann Clin Microbiol Antimicrob.

[36] Zwittink RD, Renes IB, van Lingen RA, van Zoeren-Grobben D, Konstanti P, Norbruis OF (2018). Association between duration of intravenous antibiotic administration and early-life microbiota development in late-preterm infants. Eur J Clin Microbiol Infect Dis.

[37] Sun J, Marwah G, Westgarth M, Buys N, Ellwood D, Gray PH (2017). Effects of probiotics on necrotizing enterocolitis, sepsis, intraventricular hemorrhage, mortality, length of hospital stay, and weight gain in very preterm infants: a meta-analysis. Adv Nutr.

[38] Underwood MA, Salzman NH, Bennett SH, Barman M, Mills DA, Marcobal A (2009). A randomized placebo-controlled comparison of 2 prebiotic/probiotic combinations in preterm infants: impact on weight gain, intestinal microbiota, and fecal short-chain fatty acids. J Pediatr Gastroenterol Nutr.

[39] Nandhini LP, Biswal N, Adhisivam B, Mandal J, Bhat BV, Mathai B (2016). Synbiotics for decreasing incidence of necrotizing enterocolitis among preterm neonates—a randomized controlled trial. J Matern Fetal Neonatal Med.

[40] Guney-Varal I, Koksal N, Ozkan H, Bagci O, Dogan P (2017). The effect of early administration of combined multi-strain and multi-species probiotics on gastrointestinal morbidities and mortality in preterm infants: a randomized controlled trial in a tertiary care unit. Turk J Pediatr.

[41] Grady NG, Petrof EO, Claud EC (2016). Microbial therapeutic interventions. Semin Fetal Neonatal Med.

[42] Stinson LF, Payne MS, Keelan JA (2017). Planting the seed: origins, composition, and postnatal health significance of the fetal gastrointestinal microbiota. Crit Rev Microbiol.

[43] Watkins C, Stanton C, Ryan CA, Ross RP (2017). Microbial therapeutics designed for infant health. Front Nutr.

[44] Boehm G, Moro G (2008). Structural and functional aspects of prebiotics used in infant nutrition. J Nutr.

[45] Agostoni C, Buonocore G, Carnielli VP, De Curtis M, Darmaun D, Decsi T (2010). Enteral nutrient supply for preterm infants: commentary from the European Society of Paediatric Gastroenterology, Hepatology and Nutrition Committee on Nutrition. J Pediatr Gastroenterol Nutr.

[46] Ballard O, Morrow AL (2013). Human milk composition: nutrients and bioactive factors. Pediatr Clin North Am.

[47] Medina DA, Pinto F, Ovalle A, Thomson P, Garrido D (2017). Prebiotics mediate microbial interactions in a consortium of the infant gut microbiome. Int J Mol Sci.

[48] James K, Motherway MO, Penno C, O’Brien RL, van Sinderen D (2018). Bifidobacterium breve UCC2003 employs multiple transcriptional regulators to control metabolism of particular human milk oligosaccharides. Appl Environ Microbiol.

[49] Stewart CJ, Embleton ND, Marrs ECL, Smith DP, Fofanova T, Nelson A (2017). Longitudinal development of the gut microbiome and metabolome in preterm neonates with late onset sepsis and healthy controls. Microbiome.

[50] Vos AP, van Esch BC, Stahl B, M’Rabet L, Folkerts G, Nijkamp FP (2007). Dietary supplementation with specific oligosaccharide mixtures decreases parameters of allergic asthma in mice. Int Immunopharmacol.

